# Recovery of Valuable Materials from End-of-Life Photovoltaic Solar Panels

**DOI:** 10.3390/ma16072840

**Published:** 2023-04-02

**Authors:** Dina Magdy Abdo, Ayat Nasr El-Shazly, Franco Medici

**Affiliations:** 1 Central Metallurgical Research and Development Institute, P.O. Box 87, Helwan, Cairo 11421, Egypt; 2 Department of Chemical Engineering, Materials and Environment, “Sapienza” University of Rome, Via Eudossiana 18, 00184 Roma, Italy

**Keywords:** solar panels, recycling, solid waste, physical treatment, chemical treatment

## Abstract

The disposal of end-of-life (EOL) photovoltaic solar panels has become a relevant environmental issue as they are considered to be a hazardous electronic waste. On the other hand, enormous benefits are achieved from recovering valuable metals and materials from such waste. Eventually, physical and chemical processing will become the most important stages during the recycling process. A physical treatment including crushing, grinding, and screening was achieved, and it was observed that a fine fraction of −0.25 mm had the maximum percentage of the required materials. Moreover, the optimum chemical treatment conditions were adjusted to reach the maximum recovery of silver, aluminum, and silicon. The synthesis of silicon oxide, silver oxide, alunite, and K-Alum from leachant solution was performed through a simple route. The structural and morphological properties of the prepared materials were defined by X-ray diffraction (XRD), X-ray photoelectron spectroscopy (XPS), and field emission scanning electron microscopy (FESEM).

## 1. Introduction

Recently, two major problems facing developing countries, especially in the industrial sector, are electronic solid waste management and the need for more valuable materials due to their enormous consumption [1,2,3]. One of the overgrown industries is the renewable energy sector; the generation of global photovoltaic panel (PV) electricity reached 855.7 TWh in 2020, while the installation capacity reached around 707.5 GW and is expected to reach 14.5 TW by 2050 [4]. Moreover, the global production of photovoltaic panels is expected to multiply [5]. Furthermore, for analysis, PV solar panels are grouped according to the PV module type: silicon-based “c-Si PV modules,” thin film-based “compound PV modules,” and third-generation “other PV modules (e.g., organic solar cells, dye-sensitized solar cells, perovskite solar cells). Therefore, the recycling of such modules has been identified according to the targeted recovery materials [6] displayed in Table 1.

On the other hand, solar panels’ lifetime is 25 to 30 years [7,8]. This indicates that the amount of end-of-life (EOL) solar panels will be huge; it is expected to reach 1.7–8 million tons by 2030, and its growth will reach 60–77 million tons by 2050 [9]. It will need a proper management method as it is considered hazardous electronic waste [10]. However, solar panels contain toxic metals, mainly lead [11,12]; EOL solar panels are considered rich waste because they have valuable metals, e.g., silver, aluminum, copper, tin, and silicon [13]. The recovery of such valuable metals or materials has positive impacts on both environmental and economic view points, and energy saving will be achieved, thus solving the problem of electronic waste management, and the availability of valuable materials will be realized [14]. Nowadays, crystalline silicon solar panels represent 90% of the panels market; this is due to the low prices and mature manufacturing technology [15]. The main components of such panels are: (1) an aluminum frame, (2) glass, (3) encapsulated layers (ethylene vinyl acetate (EVA) binding the solar cells together), a back sheet, and a junction box.

The most reverent method used to manage EOL solar panels is recycling aluminum frames, as separating them is easy. Additionally, some studies have recycled glass using a mechanical treatment which can separate aluminum frames efficiently from solar panels [16], while thermal treatment separates glass easily [17]. Nevertheless, few researchers have studied the recovery of the valuable metals present in EOL solar panels, while most researchers recover aluminum and then stop at this stage.

The purpose of this research is to develop a simple integrated method for EOL solar panels treatment and to recover valuable materials such as silicon oxide (SiO_2_), silver/silver oxide (Ag_2_O), and aluminum oxide compounds (Al_2_O_3_) from such hazardous waste using the chemical route. The study provides a detailed treatment process, followed by a deep characterization of the recovered materials, and, finally, an approximated cost analysis was performed to prove the study’s economic benefits.

## 2. Materials and Methods

### 2.1. Material

**Figure 1:**
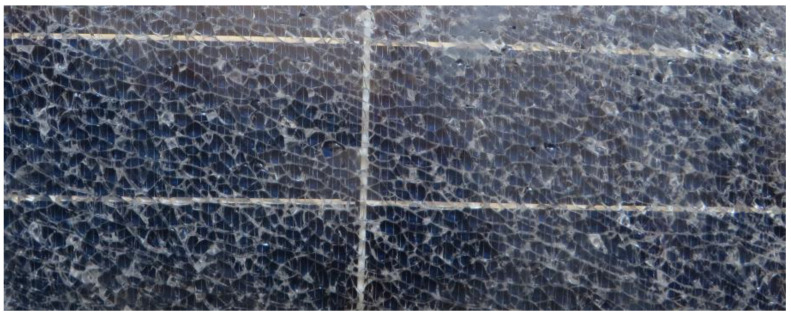
The received EOL solar panels used in the current study.

An EOL solar panel was supplied by the municipality of Celano (L’Aquila, Italy), where Europe’s largest photovoltaic park owned by a public administration is installed, where the aluminum frame was removed, as present in Figure 1. The used chemicals were of a lab grade without any further purification, including nitric acid (HNO_3_), sulfuric acid (H_2_SO_4_), hydrogen peroxide (H_2_O_2_), potassium hydroxide (KOH), sodium hydroxide (NaOH), and polyvinylpyrrolidone (PVP). Moreover, the water used during the experiment was deionized.

The procedure was performed in several stages: firstly, a physical treatment was conducted to achieve the beneficiation and concentration of valuable materials in specific fractions; secondly, chemical leaching was conducted in different steps to achieve the dissolution of the required materials; and finally, the precipitation method was conducted to synthesis the final products, followed by a characterization stage to prove the purity of the synthesized materials.

### 2.2. Physical Treatment

The EOL solar panels were cut into regular homogenous pieces of around a size of 5 × 8 cm^2^ and then crushed into small particles by a jaw crusher. This stage was followed by screening using a series of sieves of different sizes from 5.6 mm to 0.25 mm. The sample was separated into different fractions according to their particle size.

### 2.3. Chemical Leaching

The sample of particle size of −0.25 mm was mixed and divided manually to equal parts to ensure its homogeneity. For silver dissolution, 10 g of the sample was dissolved in 200 mL of 5 M HNO_3_ for 1 h at room temperature by stirring at 400 rpm using a hotplate; after that, the solution was filtrated using filter paper, and the filtrate, which was mainly silver nitrate, was saved for silver oxide precipitation. At the same time, the solid part, which was primarily rich in aluminum and silicon, was washed carefully with distillate water for further leaching with potassium hydroxide to achieve complete aluminum dissolution, where 5 g of the HNO_3_-treated sample was dissolved in 100 mL of 4M KOH at 80 °C for 2 h by stirring at 400 rpm using a hotplate, followed by a filtration stage and filtrate, which was mainly rich in aluminum, and this was saved for aluminum compound (potassium alum and alunite) precipitation. In contrast, the remaining residue was washed several times with 300 mL of hot distillate water and then dried at 60 °C overnight for the characterization of silicon compound.

### 2.4. Precipitation Method

The co-precipitation method was used as it is the most simple and economical method. For silver oxide precipitation, the following procedure was illustrated: 10 mL of 1:1 (*w*/*v* %) PVP was added gradually to 100 mL of silver nitrate filtrate while stirring, then 2 M of NaOH was added until the pH adjusted to 10; finally, 10 mL of H_2_O_2_ was added, where the brown precipitate of silver oxide started to appear, we continued stirring for 1 h, and then silver oxide was separated using a centrifuge, where it was washed several times and then dried at 60 °C overnight for a further characterization. For aluminum compound (potassium alum and alunite) precipitation: 50% of sulfuric acid solution was added to 100 mL of aluminum-rich filtrate till neutralization of the solution occurred; we continued stirring the mixture until no more white precipitate appeared, then the formed precipitate was separated using a centrifuge, where it was washed several times and then dried at 60 °C overnight, followed by calcination at 600 °C for 5 h for a further characterization. For amorphous silicon, the residue remains washed carefully to remove any remaining salts or impurities and dried for a further characterization.

### 2.5. Characterization

The chemical composition of the −0.25 mm physical treated sample was characterized by XRF (X-ray fluorescence, Axios Advanced WDXRFP analytical, Almelo, The Netherlands), while the phase of the synthesized materials was identified by XRD (X-ray diffract-meter, BrukerAXS-D8, Mannheim, Germany), and their morphology was examined by SEM (field emission scanning electron microscope; Quantafeg 250, Eindhoven, The Netherlands), and, finally, their surface structure were recorded by XPS.

## 3. Results and Discussion

### 3.1. Purification of EOL Solar Panels and Metal Separation

**Figure 2:**
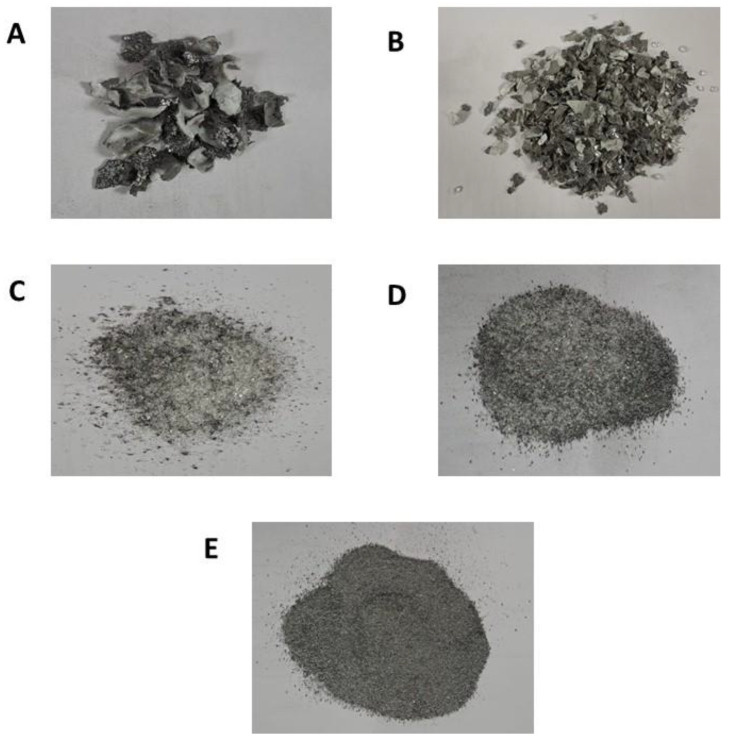
Crushed and screened sample divided into different fractions according to particle size, (**A**) fraction of +5.6 mm, (**B**) fraction of +2 mm, (**C**) fraction of +0.5 mm, (**D**) fraction of +0.25 mm, (**E**) fraction of −0.25 mm.

As discussed in the previous section, the received EOL solar panels were free from an aluminum frame; after achieving the physical treatment as discussed, different fractions were separated according to their particle size. Figure 2 shows different particle size samples, where the fraction of −0.25 mm was chosen for a further treatment. The XRF characterization of the selected fraction is explained in Table 2, where it can be observed that the required materials, including silicon, aluminum, and silver, are 70.25% (in weight) of the sample. In comparison, the other is only 29.75% (in weight).

### 3.2. Characterization of the Products

#### Structural Characterization

**Figure 3:**
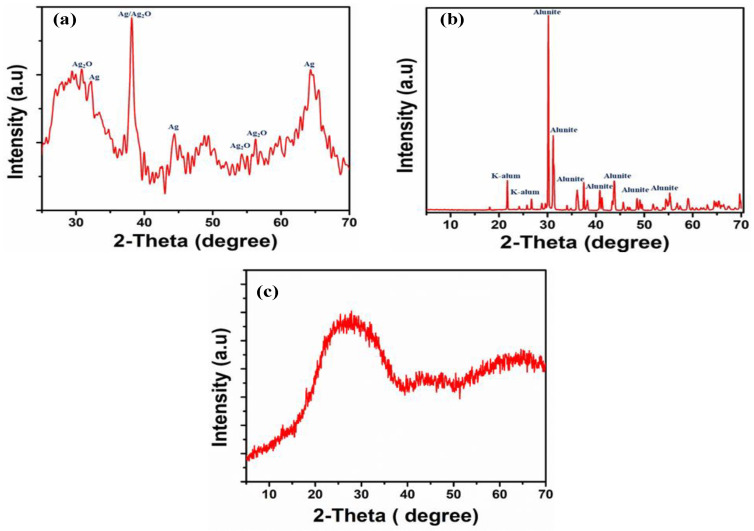
X-ray diffraction patterns of synthesized materials: (**a**) for Ag/Ag_2_O mixture, (**b**) for alunite/K-alum mixture, and (**c**) for amorphous silica.

A typical XRD pattern of the synthesized silver/silver oxide nanoparticles is shown in Figure 3a; peaks in the XRD pattern can be indexed as a face-centered cubic (fcc) structure (JCPDS, file no. 4-0783) as reported by many studies [18,19], while other peaks correspond to the presence of silver oxide nanoparticles, as mentioned by K.T. Sullivan et al. [20]. While for synthesized aluminum compounds, alunite/K-alum nanoparticle, it can be observed from Figure 3b, has most of its peaks represent the presence of alunite (KAl_3_(SO_4_)_2_(OH)_6_) and K-alum (KAl(SO_4_)_2_·12H_2_O), which matched with the results obtained from the research carried out by Lucejko et al. [21]. In contrast, the other peaks correspond to the presence of either aluminum oxide or aluminum hydroxide. In addition, as mentioned in Figure 3c, it represents the XRD pattern for amorphous silica, where it was observed that the peak of amorphous silica location shifted from 2θ = 21◦, which is due to the alkali–silica reaction [22], as silica is subjected to a reaction with potassium hydroxide for 2 h at 80 °C during the aluminum leaching stage.

**Figure 4:**
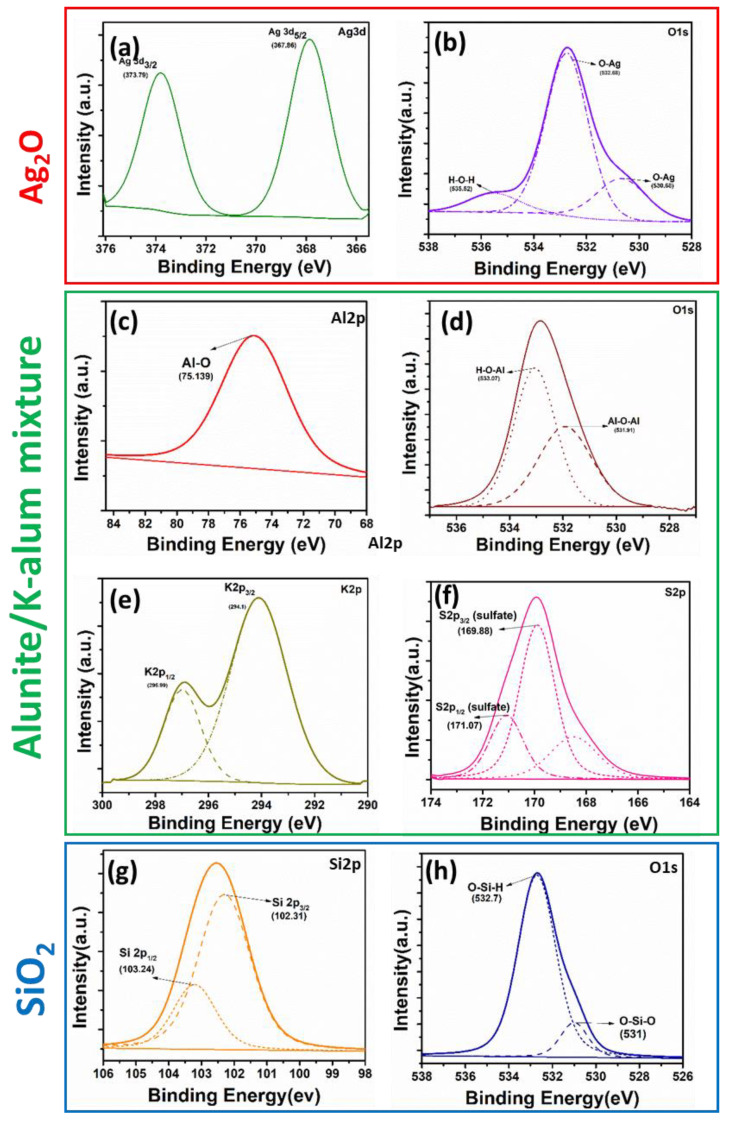
The XPS spectra of the recovered materials: (**a**,**b**) for Ag_2_O nanoparticles, (**c**–**f**) for alunite/K-alum mixture, and (**g**,**h**) for SiO_2_.

To further understand the surface chemical composition and elemental states of the recovered materials, XPS measurements were performed. Figure 4 depicts the presence of Si, Al, K, S, Ag, and O elements attributed to the mentioned materials with no other impurities found in the samples. The HR-XPS of Si 2p, Al 2p, K 2p, S 2p, Ag 3d, and O 1s are illustrated in Figure 4a–h. Typically, as shown in Figure 4a,b, for the high-resolution Ag 3d core level spectrum, the peaks at 367.86 and 373.79 eV correspond to the oxide (Ag–O) [23,24,25]. The most intense peak of the O 1s spectrum at 532.68, as shown in Figure 4 h, characterizes the formation of Ag_2_O. For the Alunite/K-alum mixture, the high-resolution Al 2p and O 1s XPS spectra (Figure 4c,d), show two peaks located at 75.139 eV for Al and 533.05 eV for O, which is within the range of the alumina binding energy [26,27]. Moreover, the sharp peaks at 296.99 and 294.1 eV are characteristic of K 2p2/3 and 2p1/2, respectively. The two peaks centered at 170.03 and 167.76 eV are also characteristics of S 2p2/3 and 2p1/2 [28], respectively (see Figure 4e,f), suggesting the presence of alunite/K-alum mixture [29]. In addition to this, as displayed in Figure 4g, for Si 2p core-level spectrum with two peaks centered at 103.24 and 102.31 eV and O 1s spectra with BEs of 531 and 532.7 eV, which demonstrate that there SiO_2_ states, as well as Si−OH, forms on the surface of SiO_2_ [30].

### 3.3. Morphological Properties

**Figure 5:**
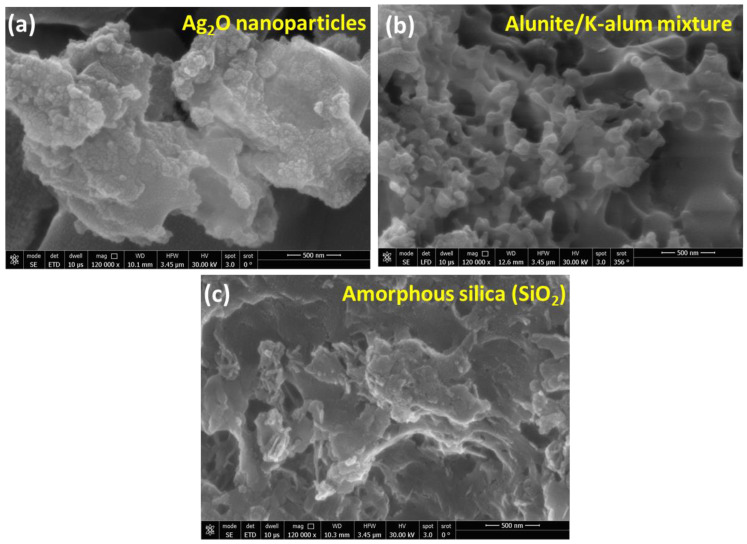
The FE-SEM images of the recovered materials: (**a**) for Ag_2_O nanoparticles, (**b**) for alunite/K-alum mixture, and (**c**) for amorphous silica (SiO_2_).

The morphological properties, as well as the microstructure of the synthesized materials, can be carried out using the FE-SEM technique. To begin with, Ag_2_O, as shown in Figure 5a, indicates that the sample mainly consists of nanoparticles with an average particle size of 20 nm. Additionally, as shown in Figure 5b for the alunite/K-alum mixture, micrographs depict a fascinating porous structure of alunite/K-alum with a uniform structure of the agglomerated particles. As shown in Figure 5c, an SEM image of amorphous silica is composed of aggregates of 700 nm with an average size of primary particles of 30 to 50 nm.

The previous characterization proved the purity and homogeneity of the recovered materials from EOL solar panels; these results demonstrate the beneficiation of such hazardous waste to be used as an advanced material source that can be used further in advanced applications [31].

## 4. Approximated Cost Analysis for the Recovery Process

The recovery of EOL photovoltaics has an appositive significant effect on the economic sector, where each component can be recycled and either reused or recovered in the form of a valuable material. Silver, aluminum, and silicon represent nearly 20% of EOL photovoltaics, but these materials have the highest proportion of EOL photovoltaics recovery benefits, representing nearly 90% of the total benefits. Thus, this study focused mainly on the beneficiation of a certain fraction of size −0.25 mm that represented almost 10% of the EOL photovoltaics; more than 70% of this fraction contains mainly silver, aluminum, and silicon [32]. Based on this study, 97% of silver, 100% of aluminum, and 100% of silicon were recovered from the EOL photovoltaic sample a fraction of the size at −0.25 mm, while the other component in different fractions will be recovered further in other studies. According to the cost of the recovered materials, the chemicals used during the process, and the approximated operating cost for one ton of EOL photovoltaic equivalent to 100 kg of a working fraction size of the −0.25 mm treatment process, an approximated cost analysis was calculated as represented in Table 3.

## 5. Conclusions

The traditional disposal of EOL photovoltlic solar panels has a negative impact on the environment, while the proposed method in this study permits the recovery of valuable materials. A simple method was performed in several stages: firstly, a physical treatment including crushing, grinding, and screening was conducted to achieve the beneficiation and concentration of valuable materials in −0.25 fractions; secondly, chemical leaching was conducted in a continuous sequence using nitric acid, then potassium hydroxide was used to achieve the dissolution of the required materials; and finally, the recovery of more than one valuable material, mainly silicon oxide (SiO_2_), silver/silver oxide (Ag_2_O), and aluminum oxide compounds, was achieved using a co-preciptation method. The recovered materials were totally characterized using different analytical methods (XRD, SEM, and XPS) that show their purity and morphology. Moreover, an approximated cost analysis was calculated, and this process proved to be profitable. Using the proposed method, the main concept of sustainability has been achieved. The current study represents a simple solution for the problems faced, such as hazardous waste, demonstrating that EOL solar panels can be beneficial from both economic and environmental points of view.

## Figures and Tables

**Table 1:** End-of-life management of PV solar panels.

Type of PVs	Module Type	Recovered Materials
First-generation “c-Si PV module”	Monocrystalline silicon Polycrystalline silicon	Si, Al, Cu, Ag, Sn, Pb
Second-generation “Compound PV module”	Cadmium telluride, copper, indium, gallium, Selenide Silicon thin film (amorphous silicon) Multijunction cells	Te, Cd, Si, Cu, Se with ZnO and InO
Third-generation “Others”	Perovskite solar cells Dye-sensitized solar cells (DSSC) Organic/polymer solar cells	Glass Semiconductor material

**Table 2:** XRF for sample of EOL solar panels used in study.

Compound	Na_2_O	MgO	Al_2_O_3_	SiO_2_	P_2_O_5_	SO_3_	K_2_O	CaO	TiO_2_	Cr_2_O_3_	NiO
Percentage %	7.508	1.492	7.90	61.90	0.616	0.50	0.089	15.40	0.234	0.22	0.061
Compound	Fe_2_O_3_	Ag_2_O	CuO	ZnO	As_2_O_3_	SnO_2_	Sb_2_O_3_	BaO	PbO	Bi_2_O_3_	SrO
Percentage %	0.87	0.45	0.11	0.038	0.052	0.259	0.887	0.946	0.22	0.065	0.21

**Table 3:** Cost analysis treatment of one ton of EOL solar on a laboratory scale.

Type	Classification	Quantity	Prices	Cost	Total Cost
Waste material	EOL solar panels	1000 kg Equivalent to	0.0053 USD/kg	USD 5.3	USD 5.3
Chemicals	Nitric acid	325 mL	60 USD/L	USD 19.2	USD 28.52
	Peroxide	100 mL = 142.2 g	6 USD/kg	USD 0.8532	
	Sodium hydroxide	10 gm	100 USD/kg	USD 1	
	Potassium oxide	220 gm	1 USD/kg	USD 0.22	
	Sulfuric acid	50 mL	125 USD/L	USD 6.25	
	polyvinylpyrrolidone	1 gm	1000 USD/kg	USD 1	
Operation cost	Collection			USD 120	USD 460
	Transportation			USD 140	
	Labors			USD 100	
	Electricity and water			USD 100	
Recovered materials	Silver oxide	0.423 kg	1000 USD/kg	USD 423	USD 802.5
	Aluminum oxide	7.9 kg	0.894 USD/kg	USD 7.062	
	Solar grade silicon	61.9 kg	6.0172 USD/kg	USD 372.46	
Net approximated Profit	Evaluation of {(recovered materials) − (waste material + chemicals + operation)}				308.7 USD/ton

Approximated prices are calculated according to updated market prices.

## Data Availability

Not applicable.
